# Use of the Wilshire Equations to Correlate and Extrapolate Creep Data of HR6W and Sanicro 25

**DOI:** 10.3390/ma11091585

**Published:** 2018-09-01

**Authors:** Vito Cedro, Christian Garcia, Mark Render

**Affiliations:** 1 National Energy Technology Laboratory, 626 Cochrans Mill Road, Pittsburgh, PA 15236, USA; 2 Mechanical Engineering Department, University of Texas Rio Grande Valley, 1201 West University Drive, Edinburg, TX 78539, USA; christian.garcia03@utrgv.edu; 3 KeyLogic Systems, Inc., 3168 Collins Ferry Road, Morgantown, WV 26505, USA; mrender@keylogic.com

**Keywords:** Wilshire equation, Larson-Miller parameter, creep strength, HR6W, Sanicro 25, time to rupture

## Abstract

Advanced power plant alloys must endure high temperatures and pressures for durations at which creep data are often not available, necessitating the extrapolation of creep life. Many methods have been proposed to extrapolate creep life, and one of recent significance is a set of equations known as the Wilshire equations. With this method, multiple approaches can be used to determine creep activation energy, increase the goodness of fit of available experimental data, and improve the confidence level of calculating long-term creep strength at times well beyond the available experimental data. In this article, the Wilshire equation is used to extrapolate the creep life of HR6W and Sanicro 25, and different methods to determine creep activation energy, region splitting, the use of short-duration test data, and the omission of very-short-term data are investigated to determine their effect on correlation and calculations. It was found that using a known value of the activation energy of lattice self-diffusion, rather than calculating $Q_{C}^{*}$ from each data set, is both the simplest and most viable method to determine $Q_{C}^{*}$. Region-splitting improved rupture time calculations for both alloys. Extrapolating creep life from short-term data for these alloys was found to be reasonable.

## 1. Introduction

Innovations in power generation require materials that are capable of withstanding high temperatures and stresses for at least 100,000 h of operation time. The high temperatures and pressures found in advanced power plants can induce creep failure in alloys. Consequently, alloys must be tested so that creep failures are prevented during service. However, creep data of new and advanced power plant alloys are often not available at the times relevant to the required design life. Various methods have been proposed to extrapolate creep life using data collected from short-duration tests. The Wilshire equations [1] are a recently-developed extrapolation method that have been used to predict long-term creep behavior of high-temperature, creep-resistant alloys [2]. Different approaches have been used to fit the Wilshire equation to creep rupture data. In this article, the Wilshire equation for times taken to rupture and the Larson-Miller parameter (LMP) equation are used to correlate and extrapolate the creep life of HR6W and Sanicro 25, which are recently commercialized, high-Cr, austenitic stainless-steel alloys. The Wilshire equation’s goodness of fit and the error of the calculated rupture times resulting from the use of different creep activation energy ($Q_{C}^{*}$) values determined by various methods are compared. This article also investigates the effect of splitting creep rupture data into above- and below-yield stress regions and examines the ability of the Wilshire equation to predict creep life greater than 10,000 h, using data with rupture times less than 10,000 h. The calculations of the Wilshire and LMP equations are compared. Finally, the effect of omitting data with rupture times less than 100 and 1000 h is investigated. The paper is constructed as follows: Section 2 gives a brief overview of the Wilshire and LMP equations; Section 3 discusses the data sets and methods used to obtain $Q_{C}^{*}$, split stress regions, and how Larson-Miller fitting parameters were obtained; Section 4 outlines the calculations and results of the study; and the final section contains conclusions.

## 2. Wilshire and Larson-Miller Parameter Equations

The classic power law equation, which is a combination of the Arrhenius [3] and Norton [4] equations, is the most established description of the creep of materials. The equation is defined as:

(1) $$\;{\dot{\varepsilon }}_{m}=A\sigma^{n}e^{-Q_{C}/RT\;}$$

where ${\dot{\varepsilon }}_{m}$ is the minimum creep rate, *A* is a material parameter, *σ* is applied stress, *n* is the stress exponent, *Q_C_* is the creep activation energy, *R* is the universal gas constant, and *T* is absolute temperature. The Monkman-Grant equation [5] is defined as:

(2) $$\;{\dot{\varepsilon }}_{m}{}^{\alpha }\;t_{r}=C_{M}$$

where *t_r_* is the time to rupture, *C_M_* is a constant, and α is the slope of $\log t_{r}$ vs. $\log{\dot{\varepsilon }}_{m}$. This can be coupled with Equation (1), with α set equal to 1, to produce the following time-to-rupture-based version of the classic power law equation.

(3) $$\;t_{r}=\frac{C_{M}}{A}\sigma^{n}e^{-Q_{C}/RT}$$

However, this equation is unreliable for predicting creep life at temperatures, stresses, and durations at which there are no experimental data available. This is due to the changing and difficult-to-predict stress exponent [6,7], which is a function of stress and temperature. Also, creep activation energy is a function of applied stress. Therefore, a creep activation energy that has been calculated in one stress region cannot be extrapolated to another. Many techniques to reliably extrapolate creep life from limited data have been proposed [8], including the newly-developed Wilshire equation and the well-established Larson-Miller parameter equation. 

### 2.1. Wilshire Equation

In 2007, Wilshire and Battenbough [1] developed a physically-based, yet fairly simple method to represent creep life as a function of applied stress and temperature in uniaxial tests. The proposed equations are:

(4) $$\;\frac{\sigma }{\sigma_{TS}}=\exp\left(-k_{1}{\left[t_{r}\exp\left(-\frac{Q_{c}{}^{*}}{RT}\right)\right]}^{u}\right)$$

(5) $$\;\frac{\sigma }{\sigma_{TS}}=\exp\left(-k_{2}{\left[{\dot{\varepsilon }}_{m}\exp\left(\frac{Q_{c}{}^{*}}{RT}\right)\right]}^{v}\right)$$

(6) $$\;\frac{\sigma }{\sigma_{TS}}=\exp\left(-k_{3}{\left[t_{\varepsilon }\exp\left(-\frac{Q_{c}{}^{*}}{RT}\right)\right]}^{w}\right)$$

where $\sigma /\sigma_{TS}$ is the ratio of applied stress to ultimate tensile strength, $t_{r}$ is time to rupture, ${\dot{\varepsilon }}_{m}$ is minimum creep rate, $t_{\varepsilon }$ is time to strain, $Q_{C}^{*}$ is creep activation energy determined at constant $\sigma /\sigma_{TS}$, *R* is the universal gas constant, *T* is absolute temperature, and $k_{1}$, *u*, $k_{2}$, *v*, $k_{3}$, and *w* are fitting constants. Applied stress can be normalized by yield strength (*σ_YS_*), but normalization by ultimate tensile strength causes the stress ratio to always lie between zero and one. The boundaries of the Wilshire equations are:

$$t_{r}\to 0,{\dot{\varepsilon }}_{m}\to \infty ,\;t_{\varepsilon }\to 0\;\mathrm{when}\;\frac{\sigma }{\sigma_{TS}}\to 1\;t_{r}\to \infty ,{\dot{\varepsilon }}_{m}\to 0,\;t_{\varepsilon }\to \infty \;\mathrm{when}\;\frac{\sigma }{\sigma_{TS}}\to 0$$

### 2.2. Larson-Miller Parameter Equation 

A common method used to extrapolate creep life is the Larson-Miller parameter equation [9], which predates the Wilshire equation by half a century. The LMP equation is:

(7) $$\;\mathrm{LMP}=T\left(\log_{10}\left(t_{r}\right)+C\right)$$

where *T* is absolute temperature, *C* is a material constant, and $t_{r}$ is time to rupture. The LMP, a function of stress, can be described by the following fitting function.

(8) $$\;\mathrm{LMP}\left(\sigma \right)=B_{0}+B_{1}\log\left(\sigma \right)+B_{2}\log{\left(\sigma \right)}^{2}+B_{3}\log{\left(\sigma \right)}^{3}+\ldots B_{m}\log{\left(\sigma \right)}^{m}$$

## 3. Methods

### 3.1. Data

The Wilshire and Larson-Miller parameter equations both require the following data from creep rupture tests: temperature, applied stress, and time to rupture. Additionally, the Wilshire equation requires the material’s ultimate tensile strength at each test temperature, and yield strength values can be used to identify creep regimes. All useful data were extracted from plots in various publications using Dagra digitization software (version 2.0.12) [10]. Heat-specific tensile strength values should be used if data were collected from specimens of multiple heats, but these values were not available from the data sources used in this work. Evans proposed a method to handle data from multiple batches [11], but this method requires more complicated analysis that is outside the scope of this study, so it was not utilized.

#### 3.1.1. HR6W

HR6W creep test data that reached failure at various temperatures were extracted from existing publications [12,13] and the manufacturer’s product bulletin [14]. In the literature, ultimate tensile strength and yield strength values were found at 700 °C [15], but not at any other relevant temperatures. American Society of Mechanical Engineers (ASME) Code Case 2684 [16] contains ultimate tensile strength and yield strength values at all temperatures at which creep test data were obtained; however, these values are not the results of experimental tests, but were established with the intent to be used in design calculations. The values found in the literature were used to develop conversion factors to adjust the ASME code case values. The ASME code case ultimate tensile strength values were multiplied by a factor of 1.021, while the yield strength values were multiplied by a factor of 1.25. It must be noted that all sources tested HR6W at slightly different compositions, which could affect the accuracy of the calculations. A total of 184 creep rupture data points were extracted; 165 had a time to rupture less than 10,000 h, and 167 had an applied stress less than the yield strength.

#### 3.1.2. Sanicro 25

For Sanicro 25, two sources of data were taken into consideration. Creep rupture data were extracted from a publication by Chai et al. [17], while ultimate tensile strength and yield strength values were obtained from the manufacturer’s product bulletin [18]. The nominal composition of the material is different in each source, which could affect the accuracy of the calculations. Because the creep test data were gathered from a single reference, the scatter of the data is low. A total of 152 creep rupture data points were extracted; 104 had a time to rupture less than 10,000 h and 75 had an applied stress less than the yield strength.

### 3.2. Wilshire Equation

Although three versions of the Wilshire equation exist, in this study, Equation (4)—the Wilshire equation for time to rupture—is used. Several methods have been used in the literature to determine $Q_{C}^{*}$. In most publications, the steps required to determine $Q_{C}^{*}$ are omitted or only generally described. In this work, $Q_{C}^{*}$ was determined using multiple methods. 

Firstly, $Q_{C}^{*}$ values were determined using Arrhenius plots. This method is the most well-known, and it is assumed (and shown to be a plausible assumption later in this work) that Wilshire used this method in his papers [1,2,19,20,21,22,23,24,25,26,27]. For this approach, existing creep data is regressed and rupture times at constant stress ratios are calculated. This work uses least squares regression and stress ratios at every tenth value (i.e., $\sigma /\sigma_{TS}=$ 0.1, 0.2, 0.3, etc.) with suitable data at each test temperature. Next, an Arrhenius plot of the natural log of time to rupture vs. the inverse absolute temperature is generated. For each stress ratio, a $Q_{C}^{*}$ value is defined as the slope of a line of best fit multiplied by the universal gas constant. An average is then taken of all $Q_{C}^{*}$ values to determine the final $Q_{C}^{*}$ value.

Secondly, $Q_{C}^{*}$ values were determined by optimizing the correlation of data on a Wilshire plot (ln[*t_r_* exp(−$Q_{C}^{*}$/RT)] vs. ln[−ln(*σ/σ_TS_*)]), as performed by Whittaker [28,29,30] and Jeffs [31]. When calculating $Q_{C}^{*}$ using an Arrhenius plot, the delta between the stress ratios influences the final average $Q_{C}^{*}$ value (e.g., $\sigma /\sigma_{TS}=$ 0.1, 0.2 and 0.3 compared to $\sigma /\sigma_{TS}=$ 0.1, 0.15, 0.2, 0.25, and 0.3). This presents a concern when calculating an average $Q_{C}^{*}$, since the stress ratios are not mathematically derived, but chosen based on the judgement of the user. Determining $Q_{C}^{*}$ by optimizing the correlation of data on a Wilshire plot eliminates concern over the variance of $Q_{C}^{*}$ at arbitrarily-chosen stress ratios. The technique used to optimize the correlation of data is generally not defined in the literature and may vary between authors. In this work, $Q_{C}^{*}$ values ranging from 1 to 500 kJ/mol were iterated with a step size of 1 kJ/mol to find the best correlation, which was quantified by the coefficient of determination (R^2^).

Thirdly, in some articles [2,20,21,22,23,24,25,28,32] the reasonableness of the calculated value of $Q_{C}^{*}$ is assessed by comparing it to an experimentally-measured or theoretically-calculated activation energy of lattice self-diffusion. If a value for the activation energy of lattice self-diffusion is known, it may be practical to use this value as $Q_{C}^{*}$ rather than calculate a value from the experimental data.

In this work, $Q_{C}^{*}$ was also determined as a function of *σ*/*σ_TS_*. This technique does not appear to be discussed in the literature.

After $Q_{C}^{*}$ has been determined, the *u* and *k_1_* fitting constants are respectively defined as the slope and exponential of the y-intercept of a best fit line on a Wilshire plot. Multiple linear regions may be visible on this plot, which would suggest that the data be separated into regions to improve the goodness of fit of the Wilshire equation. Gray and Whittaker [33] point out that Wilshire split regions using two different methods. Regions were consistently split where *σ* was equal to *σ_YS_*, but in one case, $Q_{C}^{*}$ and new fitting constants were recalculated for each region [29], while in another case, the original $Q_{C}^{*}$ value was used and only the fitting constants were recalculated for each region [1]. The latter case is known to under-predict creep life [34], and the former case, which more accurately describes the underlying physical processes [33,35,36], was used by Whittaker and Wilshire to extrapolate the creep life of Grade 22, 23 and 24 steels [35]. In this study, both region-splitting techniques are used.

### 3.3. Larson-Miller Parameter Equation

In the Larson-Miller parameter equation, the material constant *C* is generally set to 20 [9]. However, *C* can be calculated if desired. The method described by Zhu et al. [37] was used to calculate *C* in this work. The accuracy of Equation (8), the fitting function, increases with the number of terms that are used. For this study, four terms were deemed to be sufficient so the parameters *B*_0_, *B*_1_, *B*_2_ and *B*_3_ were obtained. With this information, 𝑡_𝑟_ can be estimated for any given combination of temperature and stress.

Following a method detailed by Zhu et al. [37], a matrix laboratory software (MATLAB, version 2014b) surface fitting tool was used to determine the LMP equation constants. The Larson-Miller parameter equation was arranged as:

(9) $$\;z=\frac{B_{0}+B_{1}y+B_{2}y^{2}+B_{3}y^{3}}{x}-C$$

where

$$\;z=\log\left(t_{r}\right),\;x=T,\;y=\log\left(\sigma \right)$$

## 4. Results and Discussion

### 4.1. Investigation of Multiple Methods to Determine $Q_{C}^{*}$, Region-Splitting, and the Use of Short-Term Creep Rupture Data to Extrapolate Creep Life

**Figure 1:**
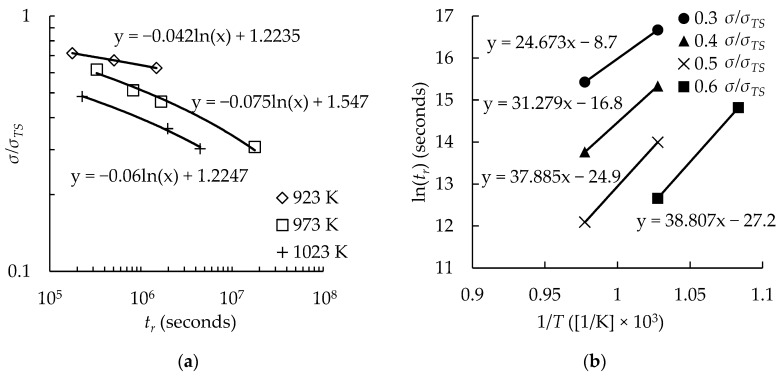
Determination of $Q_{C}^{*}$ using the traditional Arrhenius plot approach: (**a**) time to rupture as a function of the ratio of test stress to ultimate tensile strength; (**b**) Arrhenius plot of the natural log of time to rupture vs. the inverse absolute temperature.

Because this work compares different methods to determine $Q_{C}^{*}$, it is important that the methods investigated were commonly used and practical. In Wilshire’s publications [1,2,19,20,21,22,23,24,25,26,27] there is no explicit mention of the technique used to determine $Q_{C}^{*}$ except that it was calculated at constant $\sigma /\sigma_{TS}$. It was thought that a traditional Arrhenius plot was likely to be used. To test this assumption, creep rupture data were extracted from one of Wilshire’s publications [20] and a $Q_{C}^{*}$ value was calculated for comparison with the $Q_{C}^{*}$ value determined in the publication. Using the Arrhenius plot approach, $Q_{C}^{*}$ was calculated to be 230 kJ/mol; however, Wilshire calculated $Q_{C}^{*}$ as 276 kJ/mol. One figure in the publication omits all data with a time to rupture of less than 200,000 seconds, so the calculations were repeated with the same data omitted, as shown in Figure 1 and Table 1. The same value reported by Wilshire—276 kJ/mol—was calculated, which gives credibility to the assumption that Wilshire used this method.

**Figure 2:**
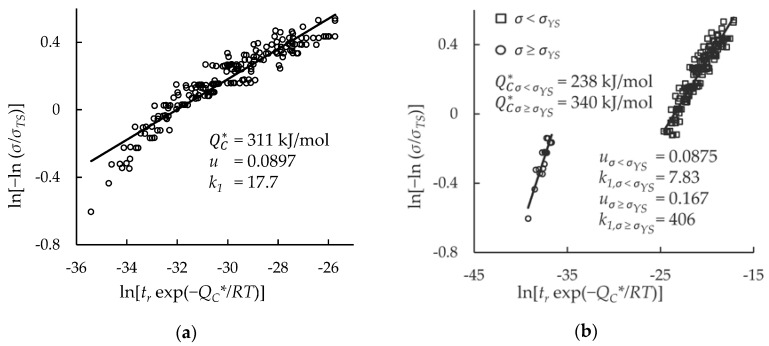

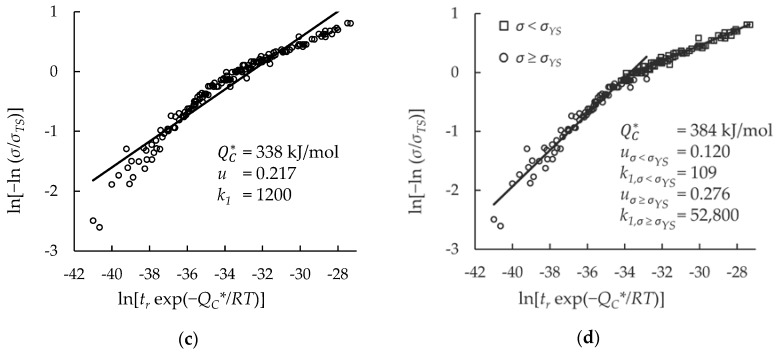
Goodness of fit of creep rupture data on Wilshire plots for: (**a**) HR6W data treated as a single region; (**b**) HR6W data split into above- and below-yield stress regions; (**c**) Sanicro 25 data treated as a single region; and (**d**) Sanicro 25 data split into above- and below-yield stress regions.

**Figure A1:**
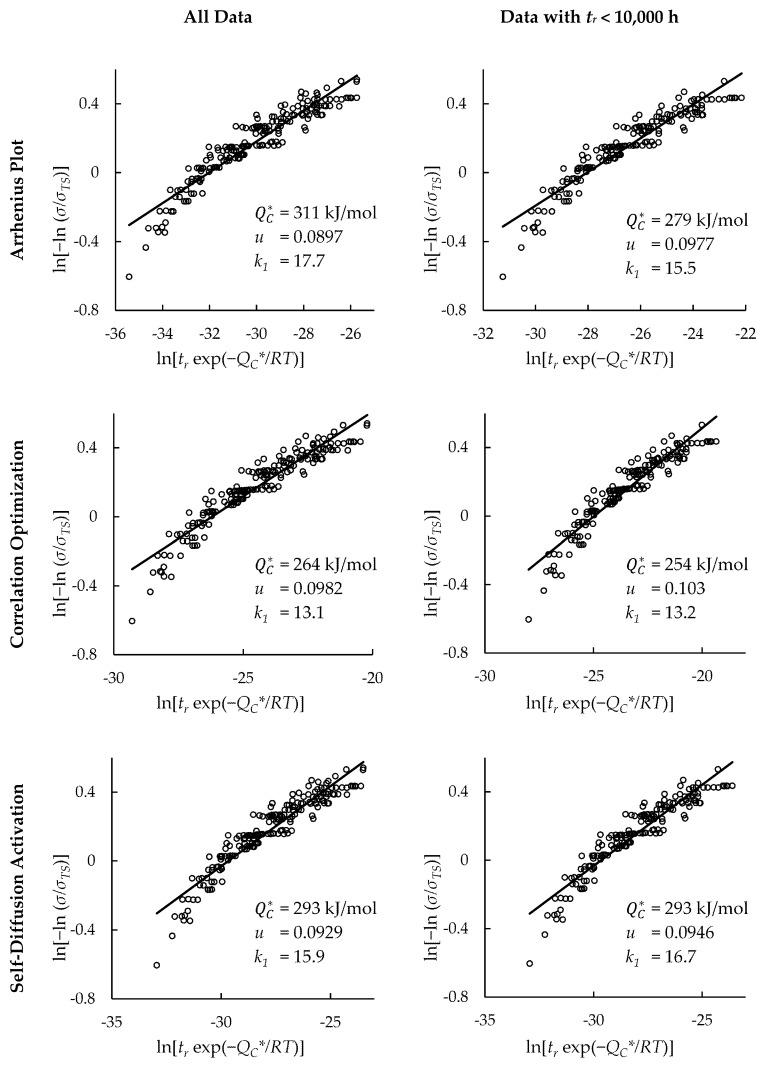
Goodness of fit of HR6W creep rupture data on Wilshire plots.

**Figure A2:**
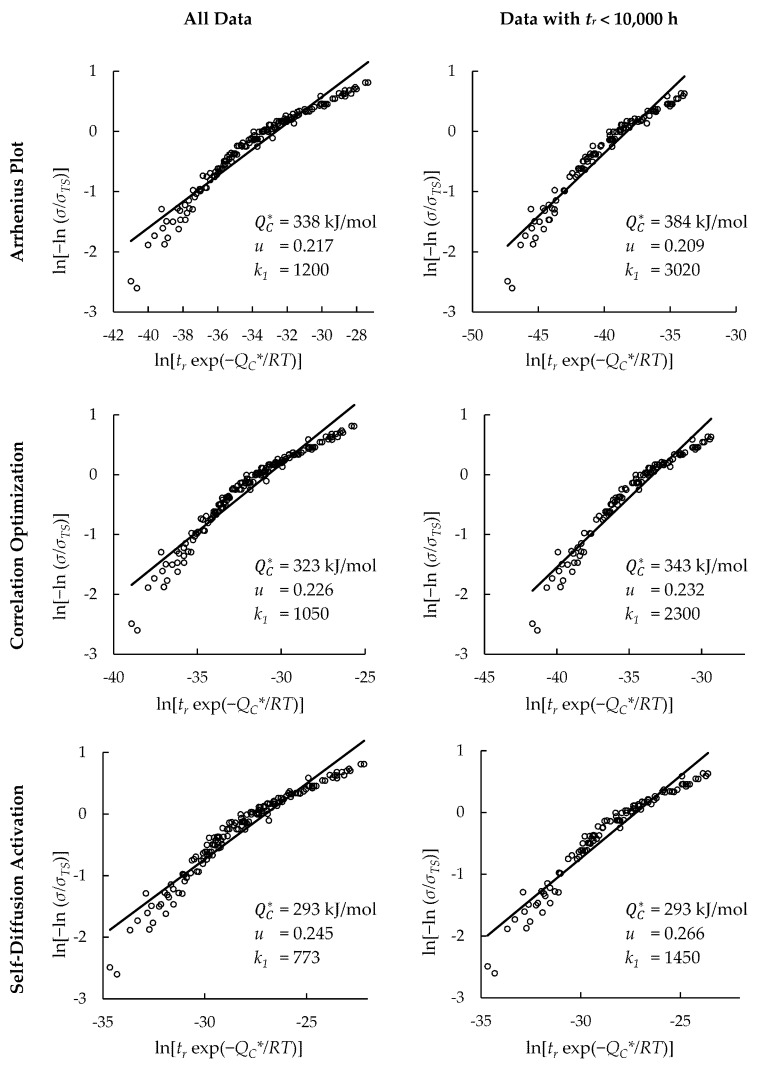
Goodness of fit of Sanicro 25 creep rupture data on Wilshire plots.

**Figure A3:**
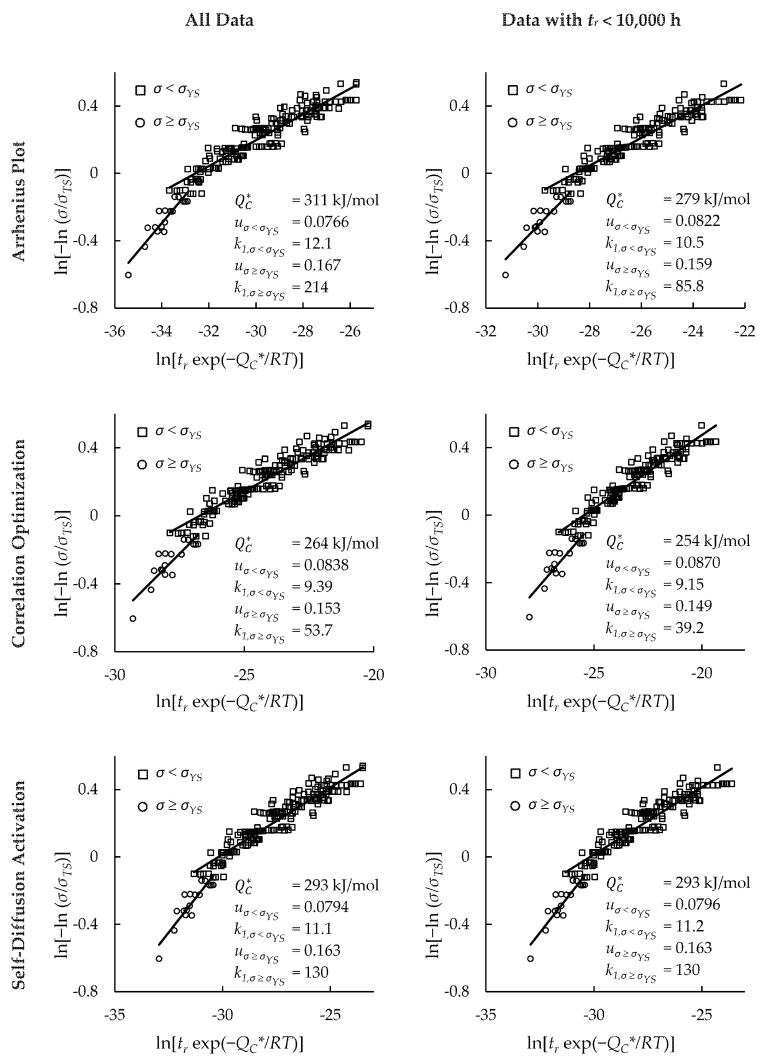
The effect of region-splitting on the goodness of fit of Wilshire plots for HR6W.

**Figure A4:**
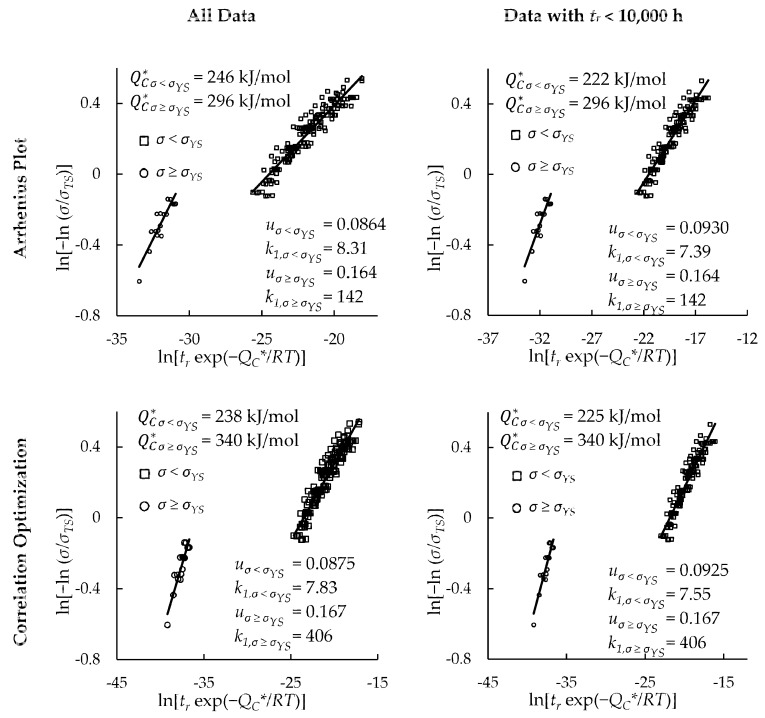
The effect of region-splitting on the goodness of fit of Wilshire plots for HR6W.

**Figure A5:**
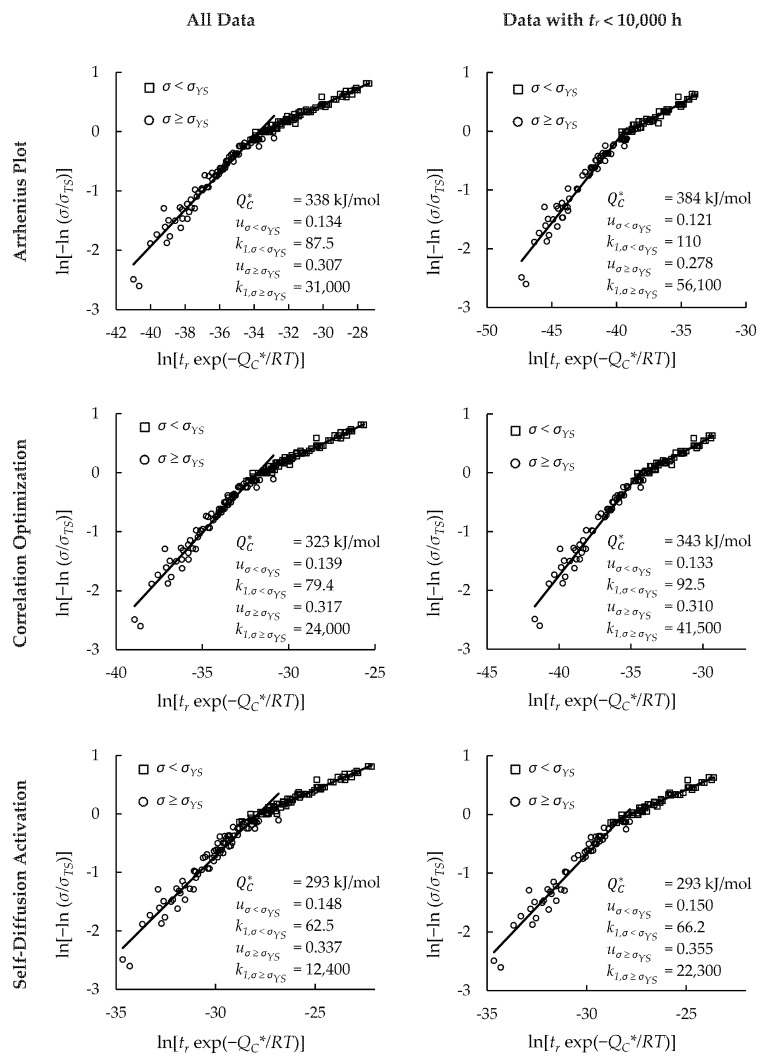
The effect of region-splitting on the goodness of fit of Wilshire plots for Sanicro 25.

**Figure A6:**
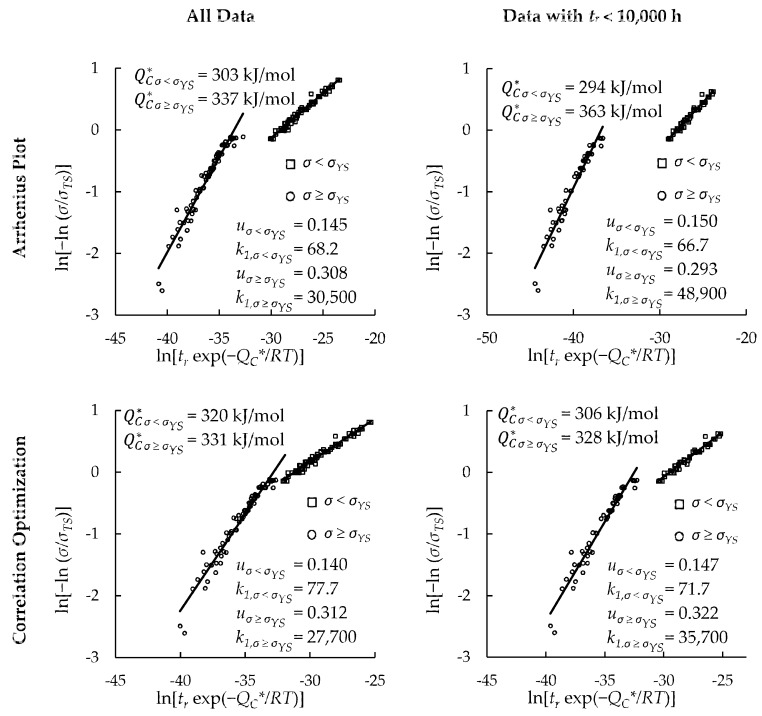
The effect of region-splitting on the goodness of fit of Wilshire plots for Sanicro 25.

Creep rupture data for HR6W and Sanicro 25 were split into two data sets for each alloy; one consisted of all data, while the other was limited to data with rupture times less than 10,000 h. The purpose of the limited data set is to show the efficacy of extrapolating short-term test data, as it has been claimed that the Wilshire equation is well-suited to do so [1,2,21,23,24,29]. For each data set, values of $Q_{C}^{*}$ were determined using Arrhenius plots (shown in Table 2), by optimizing the correlation of data on Wilshire plots (shown in Table 3), and using the activation energy of self-diffusion of austenitic stainless steels, 293 kJ/mol [38]. Wilshire plots for each case were generated to show the goodness of fit of the data. The plots for both alloys contain two clearly-defined regions that do not correlate well to the least squares regression trendlines. The Wilshire plots were split into two regions: *σ* *<* *σ_YS_* and *σ* *≥* *σ_YS_*. Representative plots are shown in Figure 2. Plots of all cases are provided as Figure A1, Figure A2, Figure A3, Figure A4, Figure A5 and Figure A6 in Appendix A.

**Figure 3:**
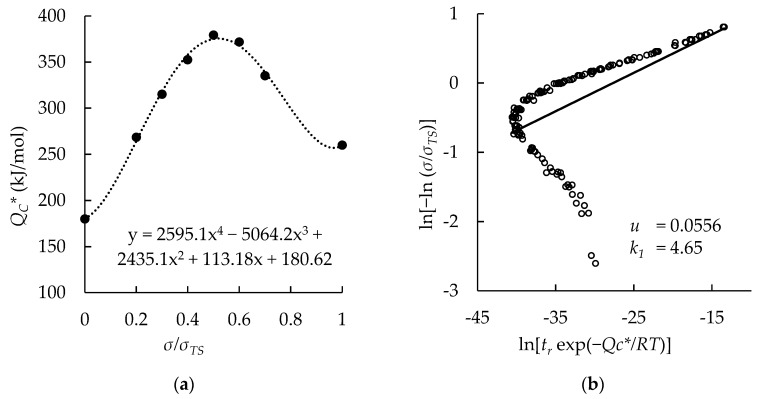
The effect of determining $Q_{C}^{*}$ as a function of the stress ratio: (**a**) 4th-order polynomial function; (**b**) Wilshire plot.

Additionally, for the Sanicro 25 data set with all data, $Q_{C}^{*}$ was determined as a function of *σ*/*σ_TS_*. $Q_{C}^{*}$ values that were previously determined using Arrhenius plots at each constant stress ratio were graphed, and $Q_{C}^{*}$ was assumed to be 180 kJ/mol at a stress ratio of 0, and 260 kJ/mol at a stress ratio of 1. A 4th-order polynomial was generated that spanned the entire domain from *σ*/*σ_TS_* = 0 to *σ*/*σ_TS_* = 1. A Wilshire plot was generated and is presented with the 4th-order polynomial in Figure 3.

The 4th-order polynomial is not a function of $Q_{C}^{*},$ so the resulting Wilshire plot bent on itself due to the rise, peak, and decline in $Q_{C}^{*}$ values as *σ/σ_TS_* increased. Therefore, the method to determine $Q_{C}^{*}$ as a function of *σ/σ_TS_* was not used in further analyses.

For all cases analyzed, time to rupture was calculated at the stress and temperature of each experimental data point. The error of the calculated rupture times in hours was measured using mean squared error (MSE), defined as:

(10) $$\;\mathrm{MSE}=\frac{\sum_{i=1}^{n}{\left(t_{r,calculated,i}-t_{r,experimental,i}\right)}^{2}}{n}$$

The Wilshire equation’s goodness of fit (quantified by R^2^) for each data set and error obtained by applying the calculated $Q_{C}^{*}$ values and fitting constants to all creep rupture data are presented in Table 4.

Splitting the data into above- and below-yield stress regions reduced the error in all cases for HR6W where $Q_{C}^{*}$ was recalculated for each region and increased the error when it was not recalculated. Region-splitting improved the error in all cases except one for Sanicro 25. When using data with rupture times less than 10,000 h to calculate $Q_{C}^{*}$, the error was reduced in all but one case for HR6W and increased in all but one case for Sanicro 25. This inconsistency may have been caused by the disproportionate quantity of creep data that ruptured at less than 10,000 h—90 percent of HR6W specimens, compared to 68 percent of Sanicro 25 specimens.

For HR6W, splitting the data and calculating $Q_{C}^{*}$ in each region using the correlation optimization method consistently resulted in the lowest error. The lowest error for Sanicro 25 was achieved by splitting the data and using the correlation optimization method to calculate a single $Q_{C}^{*}$ for the entire data set. The Arrhenius plot method never provided the lowest error—although it was close for Sanicro 25 data with rupture times less than 10,000 h split into above- and below-yield stress regions—and provided the highest error in more than half of the cases.

**Figure 4:**
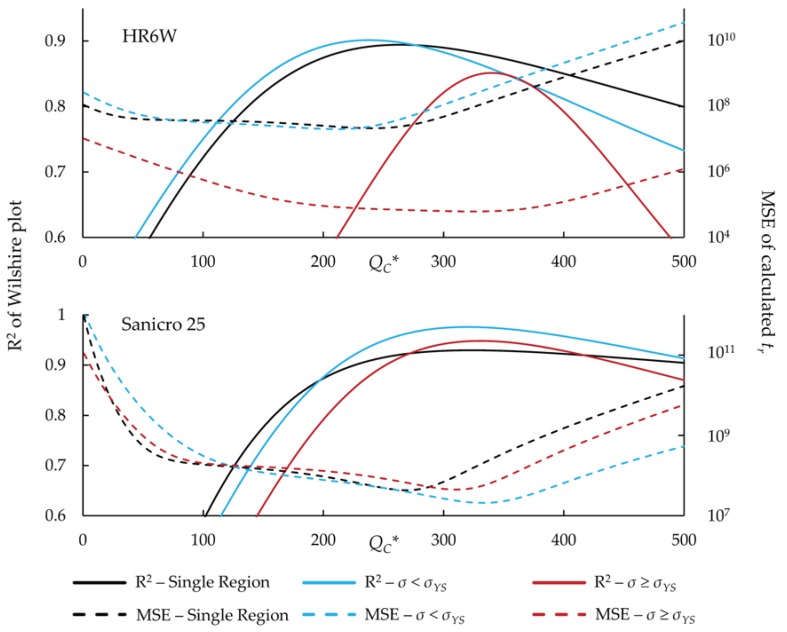
R^2^ and MSE versus $Q_{C}^{*}$ for all data.

Figure 4 shows the correlation of the Wilshire equation and error at each potential value of $Q_{C}^{*}$, which were obtained when using the correlation optimization method to determine $Q_{C}^{*}$, for each alloy. The lowest error does not occur at the highest correlation, but it is close.

**Figure 5:**
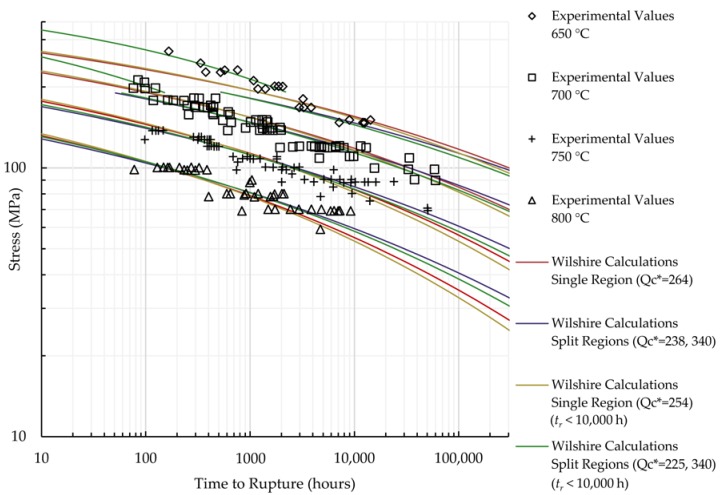
Calculations of the Wilshire equation with the lowest error for HR6W.

**Figure 6:**
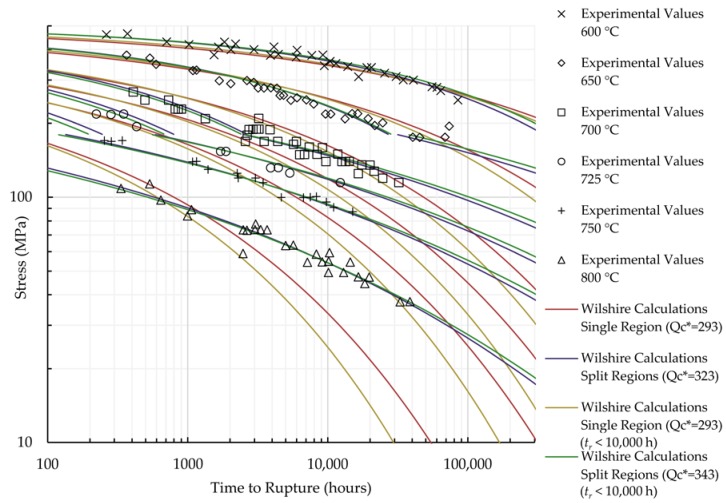
Calculations of the Wilshire equation with the lowest error for Sanicro 25.

**Figure A7:**
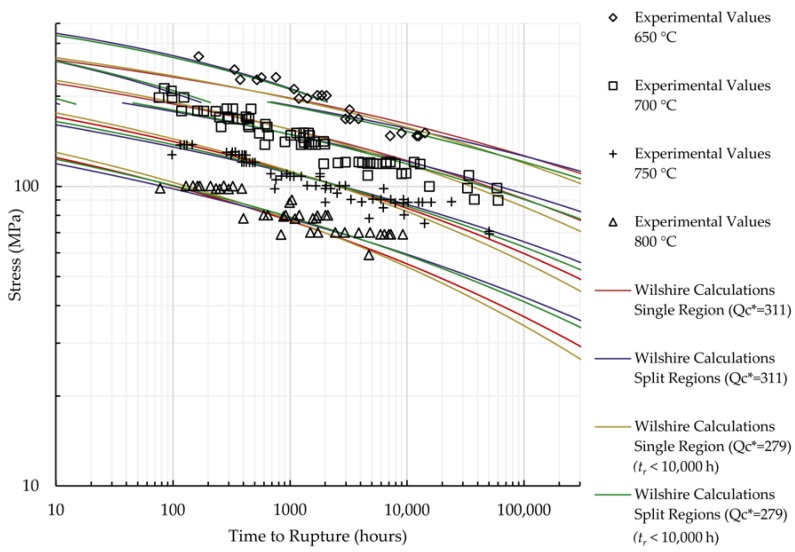
Calculations of the Wilshire equation for HR6W with $Q_{C}^{*}$, determined using Arrhenius plots.

**Figure A8:**
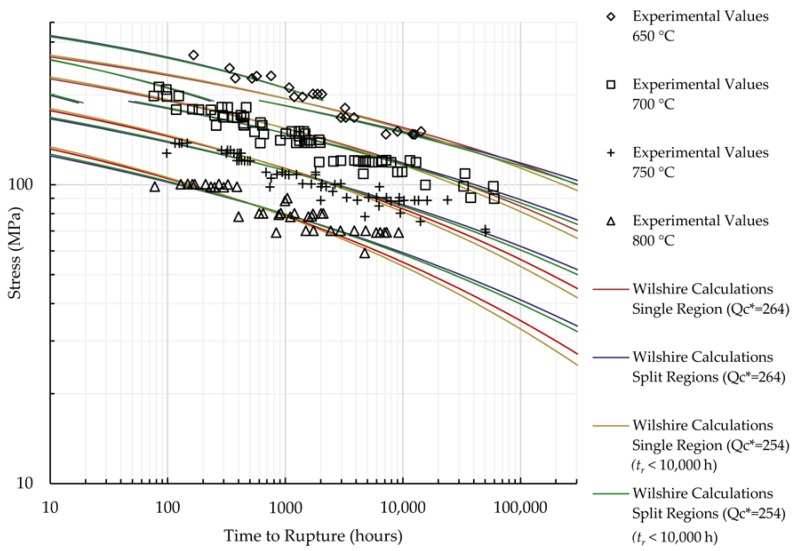
Calculations of the Wilshire equation for HR6W with $Q_{C}^{*}$, determined by optimizing the correlation of data on a Wilshire plot.

**Figure A9:**
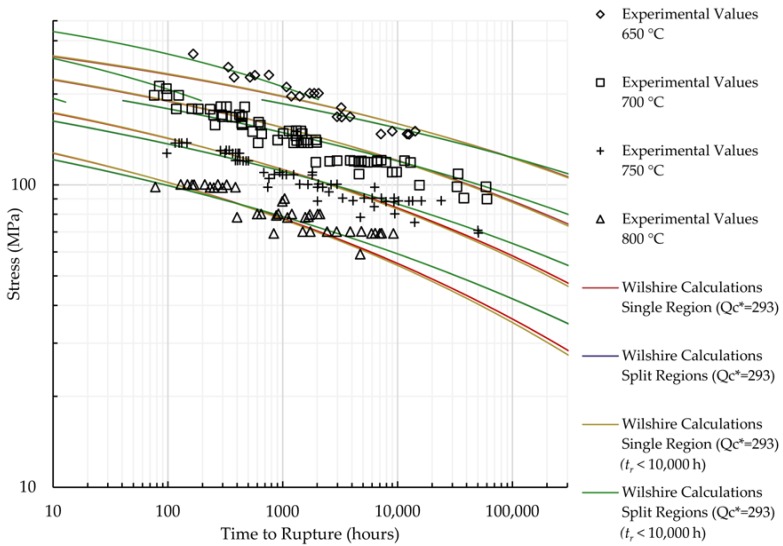
Calculations of the Wilshire equation for HR6W with the self-diffusion activation energy of austenitic stainless steels as $Q_{C}^{*}$.

**Figure A10:**
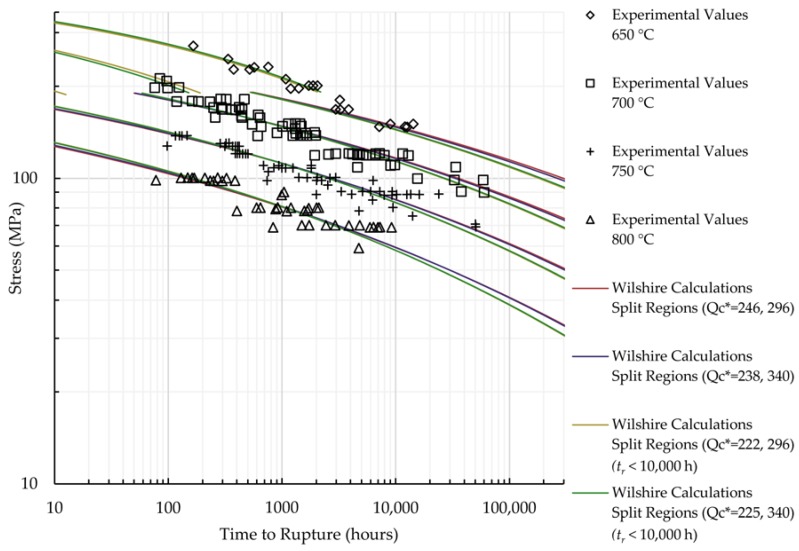
Calculations of the Wilshire equation for HR6W with $Q_{C}^{*}$ calculated in each region.

**Figure A11:**
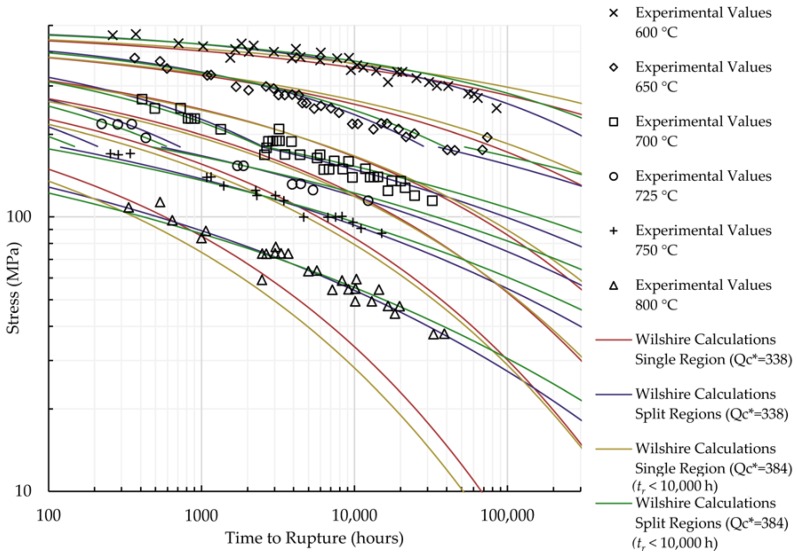
Calculations of the Wilshire equation for Sanicro 25 with $Q_{C}^{*}$, determined using Arrhenius plots.

**Figure A12:**
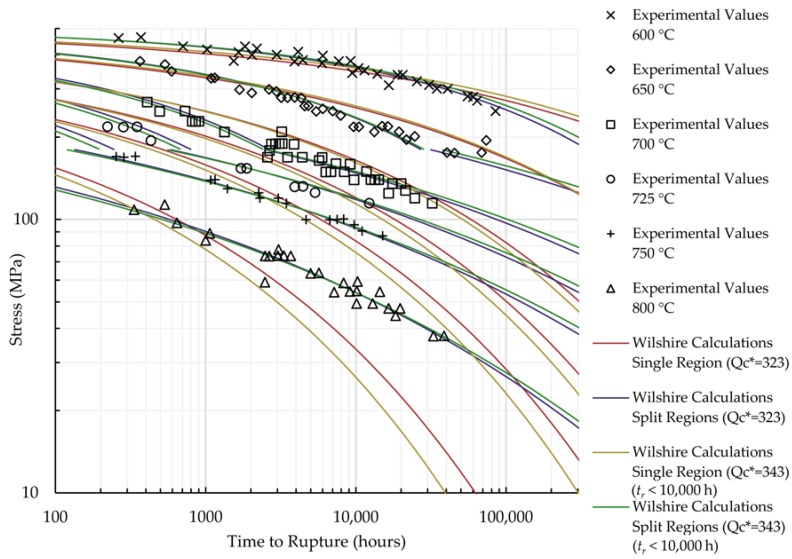
Calculations of the Wilshire equation for Sanicro 25 with $Q_{C}^{*}$, determined by optimizing the correlation of data on a Wilshire plot.

**Figure A13:**
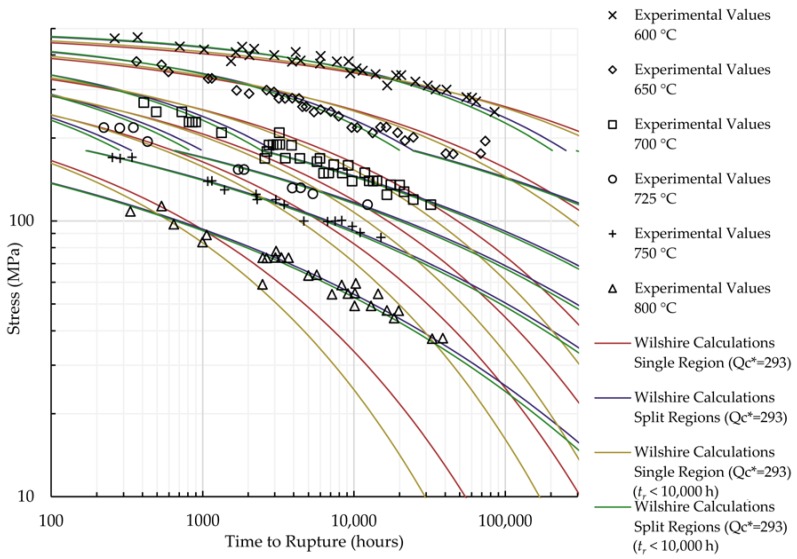
Calculations of the Wilshire equation for Sanicro 25 with the self-diffusion activation energy of austenitic stainless steels as $Q_{C}^{*}$.

**Figure A14:**
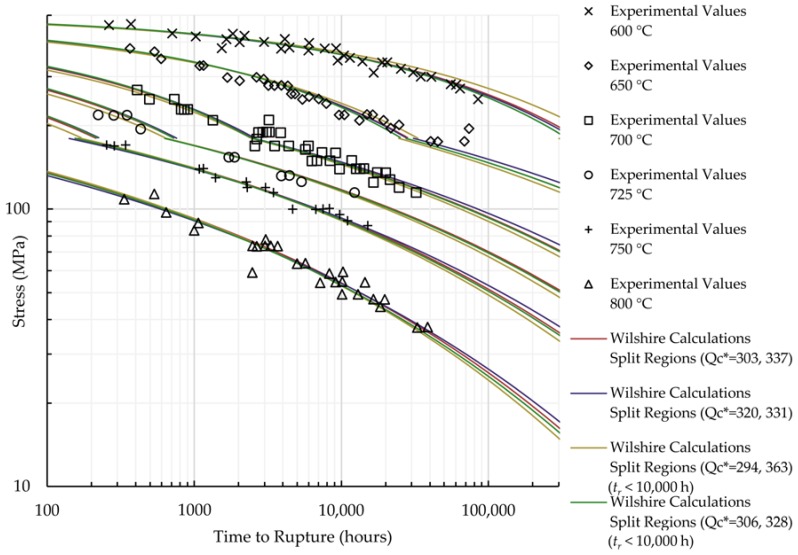
Calculations of the Wilshire equation for Sanicro 25 with $Q_{C}^{*}$ calculated in each region.

The calculations of the Wilshire equation were plotted as stress vs. time to rupture. Plots for the method that provided the lowest error for each case are shown in Figure 5 and Figure 6, while the remaining are shown in Figure A7, Figure A8, Figure A9, Figure A10, Figure A11, Figure A12, Figure A13 and Figure A14 in Appendix A. When splitting the data into above- and below-yield stress regions, the time to rupture at the transition from one region to the other is not calculated to be the same value in each region. Because of this, the split-region calculations of the Wilshire equation can yield zero or two stress values at some rupture times. The plots show the tendency of the single-region rupture stress calculations to become more conservative than the split-region calculations as time increases. Additionally, the use of data with rupture times less than 10,000 h yields visually-similar curves to those obtained using all data, which agrees with the notion that the Wilshire equation can reasonably predict creep life based on short-duration test data. 

The tendency of the Wilshire equation to over- or under-predict creep life can be quantified as the average percentage difference between the calculated and experimentally-obtained rupture times at each $Q_{C}^{*}$ value and test temperature, defined as:

(11) $$\;\mathrm{Average}\;\mathrm{Percentage}\;\mathrm{Difference}=\frac{\sum_{i=1}^{n}\left(\frac{t_{r,calculated,i}-t_{r,experimental,i}}{t_{r,experimental,i}}\right)}{n}\times 100$$

For all cases, the average percentage difference was calculated at each temperature, and the results are shown in Table 5.

For both alloys, the calculated rupture times when treating all data as a single region are generally conservative at the lowest and highest temperatures; however, at all intermediate temperatures the calculations are not conservative. This trend occurs in a less definitive manner for most, but not all cases. For Sanicro 25, the percentage differences at 700 °C, 725 °C, 750 °C and 800 °C were drastically improved by splitting the data into stress regions.

Overall, the method used to determine $Q_{C}^{*}$ has a minor effect on the goodness of fit of the Wilshire equation to the experimental data when regions are not split. This is indicated by relatively small changes to the R^2^ value when $Q_{C}^{*}$ is varied; the largest variance between R^2^ values is less than a percent. When region splitting is utilized, there is a similar trend of small changes to the R^2^ value when $Q_{C}^{*}$ is varied, except for HR6W data with applied stress greater than yield strength. In this region, the largest variance is about 15 percent. However, only nine percent of HR6W data points are in this region, which could explain the large variances. Conversely, the mean squared error of the calculated rupture times can change drastically depending on the $Q_{C}^{*}$ value, which suggests that the method used to determine $Q_{C}^{*}$ significantly impacts the calculations of the Wilshire equation. No method used to determine $Q_{C}^{*}$ consistently provided the lowest error regardless of the region-splitting technique or data set. However, the single lowest error for each alloy was calculated by splitting regions and by using a $Q_{C}^{*}$ value determined with the correlation optimization method. Additionally, the correlation optimization method provided the lowest error in all cases with split regions and half of the single region cases. Although using a self-diffusion activation energy value obtained from literature only provided the lowest error in two of the eight cases, it is the simplest method to determine $Q_{C}^{*}$ and likely accurate enough for use in many applications.

If the goal of fitting the experimental data is to minimize the mean squared error—or any other measure of error—of the calculated rupture times, it is possible to do this using a technique similar to the correlation optimization approach described and used in this work. However, a $Q_{C}^{*}$ value determined using this method may not be consistent with the scientific theory of creep.

### 4.2. Comparison of Calculations of the Wilshire and Larson-Miller Parameter Equations

**Figure A15:**
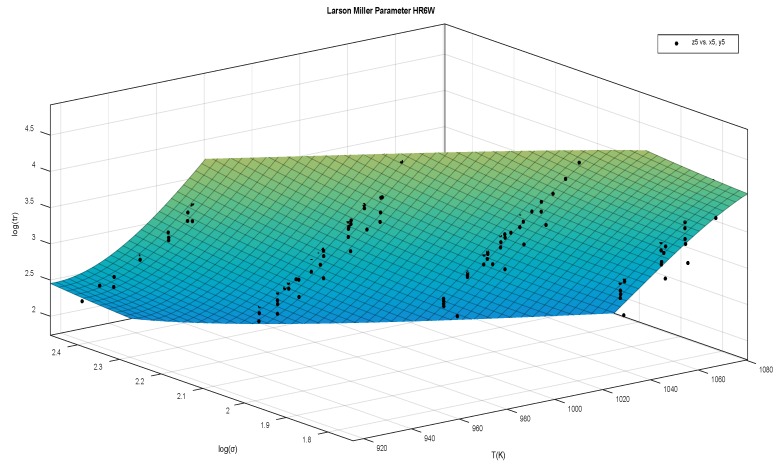
HR6W Larson-Miller parameter surface fit.

**Figure A16:**
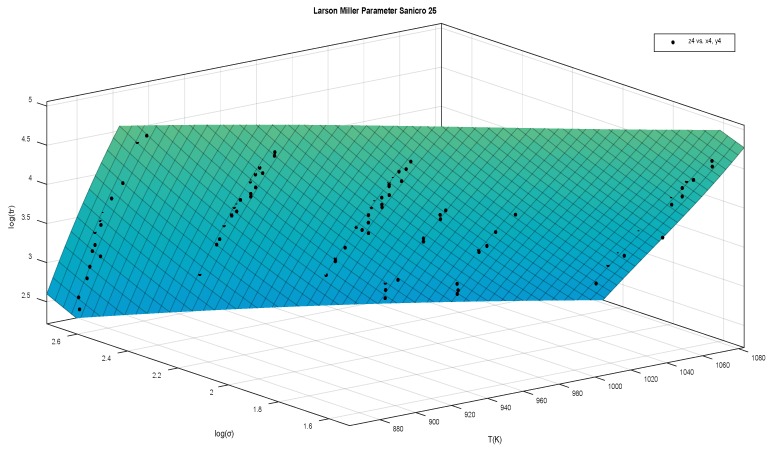
Sanicro 25 Larson-Miller parameter surface fit.

The Larson-Miller parameter equation was used to provide calculations for comparison with the calculations of the Wilshire equation. Equation (9) and the MATLAB surface fitting tool were used to correlate the experimental data to the LMP equation, and the resulting coefficients and goodness of fit are shown in Table 6. The MATLAB plots are shown as Figure A15 and Figure A16 in the Appendix A.

Time to rupture was calculated at the stress and temperature of each experimental data point using the LMP equation. The mean squared error of the calculated rupture times is compared to the lowest error obtained using the Wilshire equation with all data in Table 7. The goodness of fit and error of the Wilshire calculations are very close to those of the LMP equation.

Similar to the Wilshire equation, the tendency of the LMP equation to over- or under-predict creep life can be quantified as the average percentage difference between the calculated and experimentally-obtained rupture times, which is shown in Table 8. For both equations, the calculated rupture times at each temperature are rarely conservative.

The percentage differences of the calculated rupture time for the longest test duration of each alloy, defined as:

(12) $$\;\mathrm{Percentage}\;\mathrm{Difference}=\frac{t_{r,calculated}-t_{r,experimental}}{t_{r,experimental}}\times 100$$

are shown in Table 9. For the data point with the longest rupture time, the Wilshire equation provides a more conservative calculation than the LMP equation.

**Figure 7:**
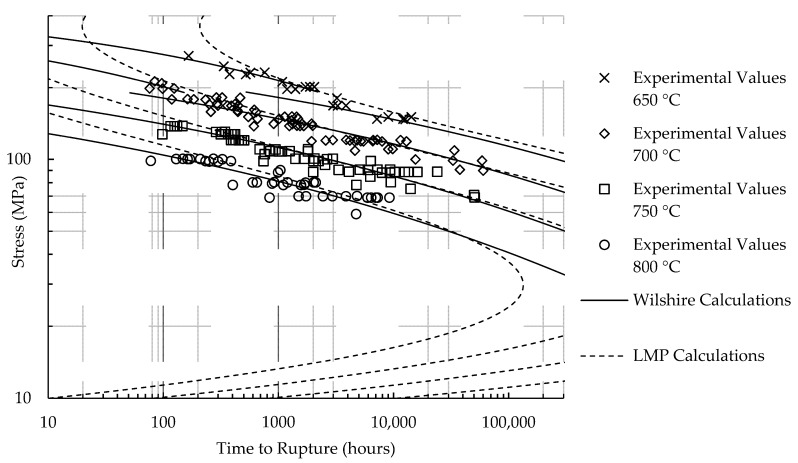
Calculated rupture times for HR6W.

**Figure 8:**
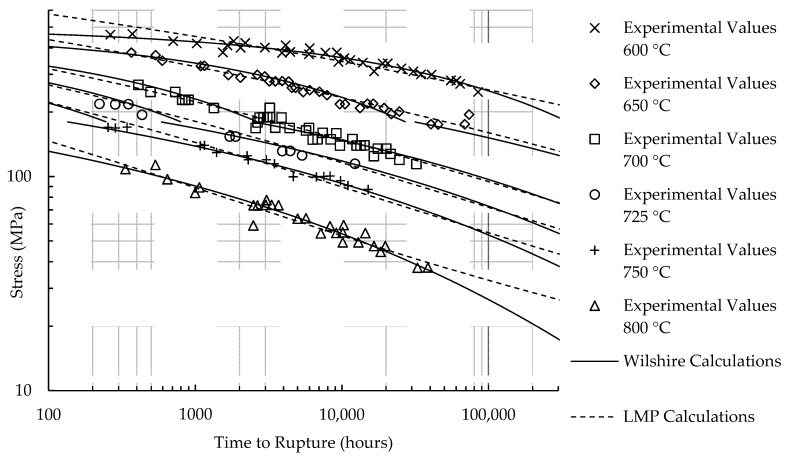
Calculated rupture times for Sanicro 25.

Calculated creep strengths for rupture at 100,000 h using the LMP and Wilshire equations are presented in Table 10. Figure 7 and Figure 8 show experimental creep data, calculations of the LMP equation, and calculations of the Wilshire equation with the $Q_{C}^{*}$ value that yielded the lowest error. The form of the LMP equation allows for multiple values of stress to be calculated at a single rupture time. Consequently, an inflection point exists in the LMP calculations for HR6W and calculated rupture times at stresses beyond the local minimum and maximum are unreasonable. The calculations of the Wilshire equation tend to be more conservative than those of the LMP equation at failure times approaching and beyond 100,000 h.

### 4.3. The Effect of Omitting Data with Rupture Times Less than 100 and 1000 h

The effect of removing data with very short rupture times (less than 1000 h) on long-term predictions of the Wilshire equation was investigated. HR6W creep rupture data were split into three data sets for comparison: all data, data with a rupture time greater than 100 h, and data with a rupture time greater than 1000 h. Sanicro 25 creep rupture data were split into two data sets: all data, and data with a rupture time greater than 1000 h (Sanicro 25 had no data with a rupture time less than 100 h). This analysis used $Q_{C}^{*}$ values determined by splitting all data into above- and below-yield stress regions and by using the correlation optimization method to calculate $Q_{C}^{*}$ for each region.

The goodness of fit of the Wilshire equation, quantified by R^2^, and the mean squared error of the calculated rupture times, obtained by applying the $Q_{C}^{*}$ and the fitting constants to all creep rupture data, are presented in Table 11.

Upon removal of data with rupture times less than 100 h, the correlation improved considerably for HR6W data that had applied stress greater than yield strength. When data with rupture times less than 1000 h were removed, only six HR6W data points with applied stress greater than yield strength were available, and a very low correlation was calculated. Small changes to the goodness of fit occurred for Sanicro 25.

The percentage differences of the calculated rupture time for the longest test duration of each alloy are shown in Table 12. The longest rupture time was 59,939 h at 700 °C for HR6W and 84,868 h at 600 °C for Sanicro 25. The percentage difference improved for HR6W data with rupture times greater than 100 h, but worsened for the other cases.

Creep strength predictions of 100,000-hour rupture times are presented in Table 13. With an exception of the HR6W data set with times to rupture greater than 1000 h, only small changes can be noticed.

**Figure 9:**
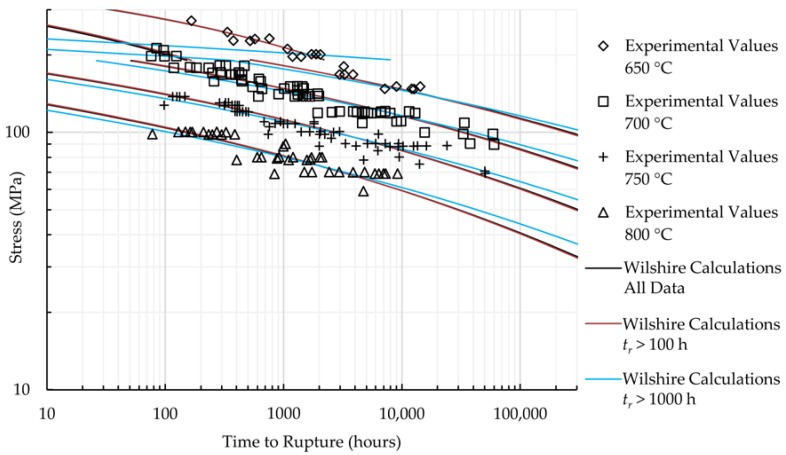
HR6W Wilshire equation calculations.

**Figure 10:**
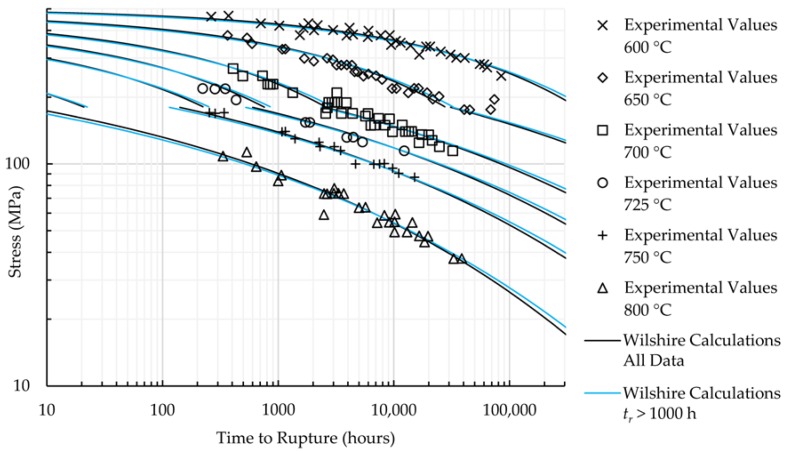
Sanicro 25 Wilshire equation calculations.

Data points at very short rupture times are sometimes removed to make calculated longer-term rupture life more conservative, but an improvement was only noticed when removing HR6W data with times to rupture less than 100 h. An insufficient amount of HR6W data with times to rupture greater than 1000 h caused poor results. No substantial improvement was observed for Sanicro 25. Figure 9 and Figure 10 provide additional insight into the effect of removing data with rupture times less than 100 and 1000 h on the predictions of the Wilshire equation.

## 5. Conclusions

This study investigated multiple methods to determine $Q_{C}^{*}$, the effect of region-splitting, the use of short-term creep rupture data to extrapolate creep life, and the omission of very-short-term creep rupture data, all of which are techniques that have been used or proposed to increase the accuracy of the Wilshire equation. Determining $Q_{C}^{*}$ by optimizing the correlation of creep rupture data on a Wilshire plot resulted in the lowest error for most cases, but using an activation energy of self-diffusion found in the literature also gave acceptable results. Splitting the data into above- and below-yield stress regions provided the lowest error for both alloys. The use of data with rupture times less than 10,000 h to extrapolate creep life was reasonable for Sanicro 25, and yielded lower mean squared errors than the use of all data for HR6W. Omitting very-short-term data improved the goodness of fit and error in one case for HR6W, but not for Sanicro 25. The Wilshire and LMP equations had a similar goodness of fit to the data and error of their calculations.

One limitation of this study was the lack of hot tensile strength data for each of the heats that comprised the data sets analyzed. Reasonable correlations of the data were obtained in some cases, but it is not known how much the data fits would have improved if tensile strength data for each heat of HR6W and Sanicro 25 had been available. 

In its basic form, the Wilshire equation is a simple method to quickly estimate long-term creep life using only three fitting constants—yet if an extensive analysis with a high level of precision is required, its complexity can be increased to improve the statistical fit of the Wilshire equation to available data. Evans [11,39,40,41,42,43,44] has proposed more sophisticated methods of fitting the Wilshire equation to complex data sets, including the handling of data collected from specimens of multiple batches [11], determining $Q_{C}^{*}$ as a function of temperature [11], statistically determining the number of stress regions [39], and utilizing additional batch characteristics [40]. The Wilshire equation is modular in that many combinations of these methods can be used, which gives it flexibility for a wide variety of applications. If only a preliminary estimate of long-term creep strength (e.g., at 100,000 h or longer design life) is needed, such as in the early stages of new alloy development, use of the Wilshire equation in its original form with $Q_{C}^{*}$ equal to the activation energy of self-diffusion would probably be sufficient. More complex analyses (which, in essence, increase the number of fitting constants) would be needed if the intent is to use the Wilshire equation for component design or for establishing long-term creep strength values for design codes, instead of the equations now used in various design codes, and which contain more than three fitting constants.

## Figures and Tables

**Table 1:** Calculated $Q_{C}^{*}$ values (kJ/mol) at each stress ratio.

Average $Q_{C}^{*}$	$\sigma /\sigma_{TS}$			
	0.3	0.4	0.5	0.6
276	205	260	315	323

**Table 2:** $Q_{C}^{*}$ values (kJ/mol) determined using Arrhenius plots.

Alloy	Data Set	Region	Average $Q_{C}^{*}$	$\sigma /\sigma_{TS}$					
				0.2	0.3	0.4	0.5	0.6	0.7
HR6W	All Data	All *σ*	311	148	235	361	501	-	-
		*σ < σ_YS_*	246	165	252	320	-	-	-
		*σ ≥ σ_YS_*	296	-	-	100	491	-	-
	*t_r_* < 10,000 h	All *σ*	279	121	221	330	444	-	-
		*σ < σ_YS_*	222	122	228	317	-	-	-
		*σ ≥ σ_YS_*	296	-	-	100	491	-	-
Sanicro 25	All Data	All *σ*	338	293	319	344	373	367	332
		*σ < σ_YS_*	303	293	324	292	-	-	-
		*σ ≥ σ_YS_*	337	-	-	294	366	361	329
	*t_r_* < 10,000 h	All *σ*	384	270	475	357	432	-	-
		*σ < σ_YS_*	294	297	322	262	-	-	-
		*σ ≥ σ_YS_*	363	-	-	-	335	358	397

**Table 3:** $Q_{C}^{*}$ values (kJ/mol) determined by optimizing the correlation of data on Wilshire plots.

Alloy	Data Set	Region	$Q_{C}^{*}$
HR6W	All Data	All *σ*	264
		σ < σ_YS_	238
		σ ≥ σ_YS_	340
	*t_r_* < 10,000 h	All *σ*	254
		*σ < σ_YS_*	225
		*σ ≥ σ_YS_*	340
Sanicro 25	All Data	All *σ*	323
		*σ < σ_YS_*	320
		*σ ≥ σ_YS_*	331
	*t_r_* < 10,000 h	All *σ*	343
		*σ < σ_YS_*	306
		*σ ≥ σ_YS_*	328

**Table 4:** Quality of fit and error.

Alloy	Data Set	Split Regions	$Q_{C}^{*}$ Determination Method	$Q_{C}^{*}$ All *σ*	$Q_{C}^{*}$ σ < *σ_YS_*	$Q_{C}^{*}$ *σ ≥ σ_YS_*	R^2^ All *σ*	R^2^ *σ* *<* *σ_YS_*	R^2^ *σ* *≥* *σ_YS_*	MSE
HR6W	All Data	No	Arrhenius Plot	311	-	-	0.887	-	-	$6.49\times 10^{7}$
			Correlation Optimization	264	-	-	0.894	-	-	$2.43\times 10^{7}$
			Self-Diffusion Activation	293	-	-	0.891	-	-	$4.08\times 10^{7}$
		Yes	Arrhenius Plot	311	-	-	-	0.877	0.835	$1.44\times 10^{8}$
			Arrhenius Plot	-	246	296	-	0.901	0.815	$2.49\times 10^{7}$
			Correlation Optimization	264	-	-	-	0.898	0.748	$3.68\times 10^{7}$
			Correlation Optimization	-	238	340	-	0.902	0.851	$2.20\times 10^{7}$
			Self-Diffusion Activation	293	-	-	-	0.887	0.810	$8.37\times 10^{7}$
	t_r_ < 10,000 h	No	Arrhenius Plot	279	-	-	0.887	-	-	$2.73\times 10^{7}$
			Correlation Optimization	254	-	-	0.889	-	-	$2.34\times 10^{7}$
			Self-Diffusion Activation	293	-	-	0.885	-	-	$3.61\times 10^{7}$
		Yes	Arrhenius Plot	279	-	-	-	0.883	0.783	$5.13\times 10^{7}$
			Arrhenius Plot	-	222	296	-	0.897	0.815	$1.99\times 10^{7}$
			Correlation Optimization	254	-	-	-	0.892	0.722	$2.49\times 10^{7}$
			Correlation Optimization	-	225	340	-	0.897	0.851	$1.97\times 10^{7}$
			Self-Diffusion Activation	293	-	-	-	0.876	0.810	$8.43\times 10^{7}$
Sanicro 25	All Data	No	Arrhenius Plot	338	-	-	0.929	-	-	$2.43\times 10^{8}$
			Correlation Optimization	323	-	-	0.930	-	-	$1.47\times 10^{8}$
			Self-Diffusion Activation	293	-	-	0.928	-	-	$5.84\times 10^{7}$
		Yes	Arrhenius Plot	338	-	-	-	0.975	0.948	$4.26\times 10^{7}$
			Arrhenius Plot	-	303	337	-	0.975	0.948	$4.42\times 10^{7}$
			Correlation Optimization	323	-	-	-	0.976	0.948	$3.50\times 10^{7}$
			Correlation Optimization	-	320	331	-	0.976	0.949	$3.84\times 10^{7}$
			Self-Diffusion Activation	293	-	-	-	0.973	0.939	$3.96\times 10^{7}$
	t_r_ < 10,000 h	No	Arrhenius Plot	384	-	-	0.934	-	-	$1.26\times 10^{9}$
			Correlation Optimization	343	-	-	0.935	-	-	$2.94\times 10^{8}$
			Self-Diffusion Activation	293	-	-	0.932	-	-	$5.18\times 10^{7}$
		Yes	Arrhenius Plot	384	-	-	-	0.949	0.945	$1.33\times 10^{8}$
			Arrhenius Plot	-	294	363	-	0.966	0.948	$1.61\times 10^{8}$
			Correlation Optimization	343	-	-	-	0.962	0.951	$1.31\times 10^{8}$
			Correlation Optimization	-	306	328	-	0.966	0.951	$1.52\times 10^{8}$
			Self-Diffusion Activation	293	-	-	-	0.965	0.947	$1.61\times 10^{8}$

**Table 5:** Average percentage difference of calculated and experimental rupture times.

Alloy	Data Set	Split Regions	$Q_{C}^{*}$ Determination Method	$Q_{C}^{*}$ All *σ*	$Q_{C}^{*}$ *σ < σ_YS_*	$Q_{C}^{*}$ *σ ≥ σ_YS_*	600 (°C)	650 (°C)	700 (°C)	725 (°C)	750 (°C)	800 (°C)
HR6W	All Data	No	Arrhenius Plot	311	-	-	-	7%	83%	-	26%	−29%
			Correlation Optimization	264	-	-	-	−24%	56%	-	29%	−11%
			Self-Diffusion Activation	293	-	-	-	-6%	72%	-	26%	−23%
		Yes	Arrhenius Plot	311	-	-	-	17%	62%	-	28%	−21%
			Arrhenius Plot	-	246	296	-	−10%	27%	-	27%	4%
			Correlation Optimization	264	-	-	-	−7%	38%	-	26%	−4%
			Correlation Optimization	-	238	340	-	−8%	22%	-	27%	8%
			Self-Diffusion Activation	293	-	-	-	7%	52%	-	26%	−15%
	t_r_ < 10,000 h	No	Arrhenius Plot	279	-	-	-	−14%	61%	-	22%	−22%
			Correlation Optimization	254	-	-	-	−28%	47%	-	22%	−14%
			Self-Diffusion Activation	293	-	-	-	−4%	70%	-	23%	−26%
		Yes	Arrhenius Plot	279	-	-	-	−45%	35%	-	24%	−12%
			Arrhenius Plot	-	222	296	-	−64%	6%	-	19%	7%
			Correlation Optimization	254	-	-	-	−54%	21%	-	21%	−5%
			Correlation Optimization	-	225	340	-	−63%	7%	-	19%	6%
			Self-Diffusion Activation	293	-	-	-	−39%	45%	-	27%	−15%
Sanicro 25	All Data	No	Arrhenius Plot	338	-	-	−13%	67%	77%	91%	26%	−48%
			Correlation Optimization	323	-	-	−19%	59%	74%	99%	32%	−45%
			Self-Diffusion Activation	293	-	-	−30%	44%	70%	116%	46%	−36%
		Yes	Arrhenius Plot	338	-	-	14%	8%	6%	18%	−9%	2%
			Arrhenius Plot	-	303	337	14%	6%	−1%	18%	−3%	9%
			Correlation Optimization	323	-	-	8%	7%	5%	24%	−6%	5%
			Correlation Optimization	-	320	331	11%	7%	3%	20%	−6%	5%
			Self-Diffusion Activation	293	-	-	−3%	6%	5%	39%	−1%	11%
	t_r_ < 10,000 h	No	Arrhenius Plot	384	-	-	48%	120%	82%	69%	0%	−66%
			Correlation Optimization	343	-	-	10%	76%	62%	74%	6%	−62%
			Self-Diffusion Activation	293	-	-	−21%	37%	43%	85%	16%	−55%
		Yes	Arrhenius Plot	384	-	-	−94%	-58%	5%	−11%	−7%	7%
			Arrhenius Plot	-	294	363	−96%	-73%	−24%	−12%	−6%	2%
			Correlation Optimization	343	-	-	−95%	-65%	−10%	−12%	−7%	2%
			Correlation Optimization	-	306	328	−96%	−71%	−21%	−12%	−6%	2%
			Self-Diffusion Activation	293	-	-	−96%	−73%	−24%	−13%	−6%	2%

**Table 6:** MATLAB calculations of the LMP coefficients and goodness of fit.

Alloy	$B_{0}$	$B_{1}$	$B_{2}$	$B_{3}$	*C*	R^2^
HR6W	$-3.89\times 10^{4}$	$1.06\times 10^{5}$	$-5.68\times 10^{4}$	9390	17.6	0.904
Sanicro 25	$4.37\times 10^{4}$	$-2.40\times 10^{4}$	$1.02\times 10^{4}$	−1800	17.8	0.940

**Table 7:** Goodness of fit and error of Wilshire and LMP calculations.

Alloy	Equation	R^2^	MSE
HR6W	LMP eq.	0.904	$2.54\times 10^{7}$
	Wilshire eq. ^1^	0.902 ^3^, 0.851 ^4^	$2.20\times 10^{7}$
Sanicro 25	LMP eq.	0.940	$3.42\times 10^{7}$
	Wilshire eq. ^2^	0.976 ^3^, 0.948 ^4^	$3.50\times 10^{7}$

^1^ Split regions with $Q_{C}^{*}$ calculated for each region by optimizing the correlation of data on a Wilshire plot; ^2^ Split regions with $Q_{C}^{*}$ calculated for the entire data set by optimizing the correlation of data on a Wilshire plot; ^3^ Correlation of data with *σ < σ_YS_*; ^4^ Correlation of data with *σ ≥ σ_YS_*.

**Table 8:** Average percentage difference of calculated and experimental rupture times.

Alloy	Calculation Method	600 °C	650 °C	700 °C	725 °C	750 °C	800 °C
HR6W	LMP eq.	-	46.5%	48.5%	-	40.1%	59.4%
	Wilshire eq. ^1^	-	−7.7%	21.7%	-	27.0%	8.3%
Sanicro 25	LMP eq.	13.6%	3.7%	7.1%	27.6%	3.7%	−0.2%
	Wilshire eq. ^2^	8.2%	6.9%	5.1%	23.8%	−6.2%	4.5%

^1^ Split regions with $Q_{C}^{*}$ calculated for each region by optimizing the correlation of data on a Wilshire plot; ^2^ Split regions with $Q_{C}^{*}$ calculated for the entire data set by optimizing the correlation of data on a Wilshire plot.

**Table 9:** Percentage errors of the calculated rupture times for the longest test durations for the Wilshire and LMP equations.

Alloy	Calculation Method	Percentage Difference
HR6W	LMP eq.	34.2%
	Wilshire eq. ^1^	23.2%
Sanicro 25	LMP eq.	37.2%
	Wilshire eq. ^2^	23.7%

^1^ Split regions with $Q_{C}^{*}$ calculated for each region by optimizing the correlation of data on a Wilshire plot; ^2^ Split regions with $Q_{C}^{*}$ calculated for the entire data set by optimizing the correlation of data on a Wilshire plot.

**Table 10:** Calculated creep strength for rupture at 100,000 h (MPa).

Alloy	Calculation Method	600 °C	650 °C	700 °C	725 °C	750 °C	800 °C
HR6W	LMP eq. solution #1	-	836	977	-	1110	1240
	LMP eq. solution #2	-	120	87.5	-	62.2	37.2
	LMP eq. solution #3	-	11.1	13.0	-	16.1	24.1
	Wilshire eq. ^1^	-	113	86.0	-	60.6	40.6
Sanicro 25	LMP eq.	254	160	94.7	71.8	54.6	32.8
	Wilshire eq. ^2^	252	152	96.9	72.7	53.3	26.6

^1^ Split regions with $Q_{C}^{*}$ calculated for each region by optimizing the correlation of data on a Wilshire plot; ^2^ Split regions with $Q_{C}^{*}$ calculated for the entire data set by optimizing the correlation of data on a Wilshire plot.

**Table 11:** The effect of removing very-short-term test data on goodness of fit and error.

Alloy	Data set	# of Data Points	$Q_{C}^{*}$ *σ < σ_YS_*	$Q_{C}^{*}$ *σ ≥ σ_YS_*	R^2^ *σ <* *σ_YS_*	R^2^ *σ ≥* *σ_YS_*	MSE
HR6W	All Data	184	238	340	0.902	0.851	$2.20\times 10^{7}$
	*t_r_* > 100 h	177	234	326	0.905	0.886	$2.15\times 10^{7}$
	*t_r_* > 1000 h	106	252	177	0.867	0.084	$7.04\times 10^{7}$
Sanicro 25	All Data	152	320	331	0.976	0.949	$3.84\times 10^{7}$
	*t_r_* > 1000 h	129	334	331	0.978	0.943	$4.85\times 10^{7}$

**Table 12:** Calculated rupture time for the longest test duration percentage differences.

Alloy	Data set	Percentage Difference
HR6W	All Data	23.2%
	*t_r_* > 100 h	17.9%
	*t_r_* > 1000 h	37.9%
Sanicro 25	All Data	32.8%
	*t_r_* > 1000 h	43.7%

**Table 13:** Calculated creep strength for rupture at 100,000 h (MPa).

Alloy	Data set	600 °C	650 °C	700 °C	725 °C	750 °C	800 °C
HR6W	All Data	-	113	86.0	-	60.6	40.6
	*t_r_* > 100 h	-	113	85.4	-	60.2	40.3
	*t_r_* > 1000 h	-	116	89.5	-	64.3	44.1
Sanicro 25	All Data	255	151	96.4	72.3	53.0	26.5
	*t_r_* > 1000 h	259	153	98.5	74.3	54.8	27.7
